# New Treatment Strategies of Depression: Based on Mechanisms Related to Neuroplasticity

**DOI:** 10.1155/2017/4605971

**Published:** 2017-04-11

**Authors:** Yu-Jhen Huang, Hsien-Yuan Lane, Chieh-Hsin Lin

**Affiliations:** ^1^Department of Psychiatry and Brain Disease Research Center, China Medical University Hospital, Taichung, Taiwan; ^2^Graduate Institute of Biomedical Sciences, China Medical University, Taichung, Taiwan; ^3^Department of Psychology, College of Medical and Health Sciences, Asia University, Taichung, Taiwan; ^4^Department of Psychiatry, Kaohsiung Chang Gung Memorial Hospital, Chang Gung University College of Medicine, Kaohsiung, Taiwan; ^5^Center for General Education, Cheng Shiu University, Kaohsiung, Taiwan

## Abstract

Major depressive disorder is a severe and complex mental disorder. Impaired neurotransmission and disrupted signalling pathways may influence neuroplasticity, which is involved in the brain dysfunction in depression. Traditional neurobiological theories of depression, such as monoamine hypothesis, cannot fully explain the whole picture of depressive disorders. In this review, we discussed new treatment directions of depression, including modulation of glutamatergic system and noninvasive brain stimulation. Dysfunction of glutamatergic neurotransmission plays an important role in the pathophysiology of depression. Ketamine, an N-methyl-D-aspartate (NMDA) receptor antagonist, has rapid and lasting antidepressive effects in previous studies. In addition to ketamine, other glutamatergic modulators, such as sarcosine, also show potential antidepressant effect in animal models or clinical trials. Noninvasive brain stimulation is another new treatment strategy beyond pharmacotherapy. Growing evidence has demonstrated that superficial brain stimulations, such as transcranial magnetic stimulation, transcranial direct current stimulation, cranial electrotherapy stimulation, and magnetic seizure therapy, can improve depressive symptoms. The antidepressive effect of these brain stimulations may be through modulating neuroplasticity. In conclusion, drugs that modulate neurotransmission via NMDA receptor and noninvasive brain stimulation may provide new directions of treatment for depression. Furthermore, exploring the underlying mechanisms will help in developing novel therapies for depression in the future.

## 1. Introduction

Major depressive disorder (MDD) is a severe major mental disorder. The lifetime prevalence of major depressive disorder is high, around 16.9% in the United States [1]. In addition to potential suicidal risk, depression leads to functional impairment which causes burden of patients, their families, and the society. In WHO report, depressive disorder is the ninth leading cause of functional disability-adjusted life years (DALYs) and the first leading cause in years lost due to disability (YLD) in 2012 [2]. However, treatment outcome of depression is suboptimal. The use of currently available antidepressants is limited by their side effects, slow response, and inadequate treatment efficacy [3]. Full remission is difficult to be achieved. Patients may still suffer from residual depressive symptoms and cannot return to their premorbid functional level. In SART∗D study, the remission rate was approximately 30% in first-line antidepressant treatment and the overall cumulative remission rate after receiving 4 step treatment was only 67% [4]. In a meta-analysis study, the overall pooled response rate of antidepressant treatment augmented with atypical antipsychotics was only 44.2% [5].

In addition to neurotransmission theory of depression, disrupted signalling pathway and neuroplasticity also play key roles in the pathophysiology of depression. Reduced neurotropic factor expressions and altered functional connectivity of neurocircuitry are found in depression [6], and these may be the new therapeutic target in the treatment of depression. In fact, current antidepressants may exert their antidepressive effect by increasing neural plasticity [7, 8]. Chronic administration of fluoxetine can enhance synaptic plasticity and increase postsynaptic spine density [9]. Therefore, novel treatment strategies are being developed to fulfill the need in the treatment of depressive disorder.

## 2. Modulating Glutamatergic System in the Treatment of Depression

Investigation of the relationship between glutamatergic system and depression begins from N-methyl-D-aspartate (NMDA) receptor. The function of NMDA receptor plays an important role in long-term potentiation (LTP), which is the neural basis of memory [10] and pathophysiology of anxiety and depressive disorder [11]. Furthermore, chronic treatments with conventional antidepressants that target the monoamine system can alter the NMDA receptor function [12]. Dysfunction of glutamatergic neurotransmission is found in patients with MDD [13]. Therefore, glutamatergic system is thought to be another keystone in the pathophysiology of depression. Compounds acting on the glutamatergic system, especially via NMDA receptor, may be potential novel antidepressants.

### 2.1. Ketamine and Other Nonselective NMDA Receptor Antagonists

Since increased activity of glutamatergic neurotransmission was found in depression and some conventional antidepressants antagonized NMDA receptor activity [14], NMDA receptor antagonist was first investigated as potential antidepressant [15]. Ketamine, one of the NMDA receptor antagonists, has rapid antidepressive effects in clinical studies [16–18]. A single subanesthetic (0.5 mg/kg) dose of ketamine over 40-minute IV infusion can improve depressive symptoms in patients with MDD [17, 19]. The response rate of a single-dose ketamine for the treatment of depression is about 50~70% [16, 17]. The antidepressant effect occurs in 4 hours after 40-minute IV infusion of ketamine and can last for 3–7 days after administration [20]. Clinically, ketamine also improves depressive symptoms in depressive patients resistant to electroconvulsive therapy (ECT) and attenuates suicidal ideation [19]. In addition to IV injection of ketamine, intranasal ketamine is another safe route for treating depression. Intranasal ketamine has been used in the treatment of chronic pain [21] and migraine with prolonged aura [22]. In a randomized, double-blind, crossover study, intranasal ketamine could improve depressive symptoms in patients with major depressive disorder at 24 hours after receiving ketamine [23].

The long-term antidepressant effect of ketamine is still under investigation. One study found that only 27% responders to a single dose of ketamine could maintain their antidepressant effect for 28 days [24]. Therefore, repeated infusion may be needed for maintaining the antidepressant effect of ketamine. In one repeated infusion trial, the overall response rate was 70.8% after receiving IV infusions of ketamine for 6 times over 12 days. Among responders, median time to relapse was 18 days after the last infusion [25]. Although several clinical studies also showed antidepressant effects of repeat-dose ketamine infusion, the sample size of these studies were small (the largest trial only enrolled 24 patients) [25–27]. No protracted adverse effects were found in these repeated dose studies [19].

Ketamine is a mixture of two isoforms, R (−) ketamine and S (+) ketamine. As analgesics, S (+) ketamine is about three to four times more potent than R (−) ketamine [28, 29]. However, S (+) ketamine has more psychotomimetic effect [30] and is associated with more cerebral and systemic hemodynamic side effect compared with R (−) ketamine [31]. Both isomers of ketamine had rapid antidepressant effect in mice model of depression [32]. Attenuated depression-like behavior in tail suspension test, forced swimming test, and 1% sucrose preference test was noted 27 to 48 hours after a single dose of ketamine injection (both isomers? Yes). However, only R (−) ketamine had a long-acting antidepressant effect. Decreased depression-like behavior was still noted in tail suspension test and forced swimming test at day 7 after injection of R (−) ketamine [32]. In another animal study, both ketamine isomers decreased depression-like behavior at 30 minutes and 24 hours after injection, but only R (−) ketamine showed antidepressant effect at 48 hours after injection [33]. In a mice study conducted by Yang et al., both isomers could improve depressive symptoms at 6-7 days after a single injection, but R (−) ketamine was significantly more potent than S (+) ketamine in antianhedonia and antidepressant effect [34].

**Figure 1:**
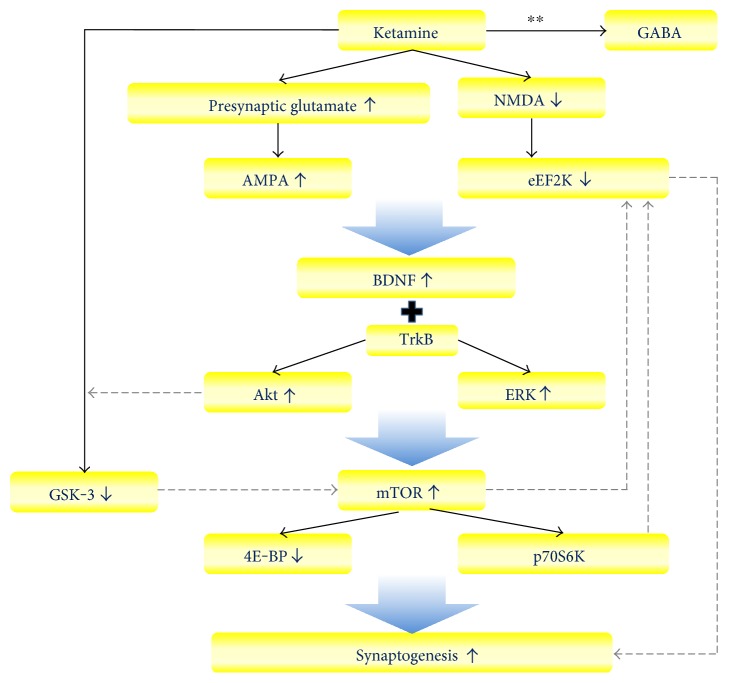
The hypothesized mechanism of ketamine's antidepressant effect. Ketamine increases AMPA receptor activities and suppresses NMDA receptor activities, which lead to activation of BDNF activity. The BDNF-TrkB signalling activates Akt and ERK pathways, which stimulate mTOR signalling. mTOR signalling increases synaptic protein synthesis by inhibiting 4E-BP and phosphorylating p70S6K. Ketamine also inhibits GSK-3 activity and may interact with GABA. ^∗^Dash line: the interaction was known in other studies, but it is still unclear in the mechanism of ketamine's antidepressant effect. ^∗∗^The interaction is still unclear because the study results are inconsistent. AMPA: *α*-amino-3-hydroxy-5-methyl-4-isoxazolepropionic acid; NMDA: N-methyl-D-aspartate; eEF2K: elongation factor-2 kinase; BDNF: brain-derived neurotrophic factor; TrkB: tropomyosin receptor kinase B; ERK: extracellular signal-regulated kinases; mTOR: mammalian target of rapamycin; 4E-BP: 4E binding proteins; p70S6K: p70 S6 kinase; GSK-3: glycogen synthase kinase-3; GABA: *γ*-aminobutyric acid.

The underlying mechanism of ketamine's antidepressant effect is complex (See Figure 1). One hypothesized model is that ketamine increases presynaptic glutamate release, resulting in activation of Akt and extracellular signal-regulated kinase (ERK) signalling, which in turn stimulates mammalian target of rapamycin (mTOR) signalling [35, 36]. Then, activated mTOR pathway increases downstream synaptic protein synthesis by phosphorylating p70 S6 kinase (p70S6K) and inhibiting 4E binding proteins (4E-BP) [35, 36]. A low dose of ketamine (10 mg/kg) can rapidly activate mTOR signalling pathway in the prefrontal cortex of rats [37]. Besides, this mTOR activation only occurs at subanesthetic doses of ketamine (5 to 10 mg/kg), but not at a higher anesthetic dose of ketamine [37]. In addition to activating mTOR signalling, ketamine also stimulates Akt and ERK pathways rapidly. Furthermore, inhibition of the Akt and ERK signalling blocks ketamine's effect on mTOR activation [37].

Ketamine also modulates mTOR signalling by increasing brain-derived neurotrophic factor (BDNF) activity [36]. In an animal study, ketamine's antidepressant effect was blocked in BDNF conditional deletion mutant [38]. Ketamine increases BDNF activity by stimulating *α*-amino-3-hydroxy-5-methyl-4-isoxazolepropionic acid (AMPA) receptor, which leads to activity-dependent release of BDNF [39]. Ketamine also suppresses NMDA receptor activities and then inhibits downstream eukaryotic elongation factor-2 kinase (eEF2K), which relieves its inhibition upon BDNF translation [40]. Then, BDNF interacts with tropomyosin receptor kinase B (TrkB) receptors and Akt and ERK signalling, which increase downstream mTOR activation [36, 37].

All the changes described above increase synaptogenesis and contribute to the rapid antidepressant effect of ketamine [35, 36, 38]. P70S6K and 4E-BP1 phosphorylation were increased after ketamine administration [35]. A single dosage of ketamine can increase levels of postsynaptic density proteins, including activity-regulated cytoskeletal protein (Arc), glutamate-AMPA receptor-1 (GluR1), postsynaptic density protein-95 (PSD95), and synapsin I [37]. These changes occur within 1-2 hours after ketamine infusion and can persist to 72 hours after ketamine administration [36, 37]. The effect of ketamine to synaptogenesis can be proved by increasing the number of mature mushroom-shaped spines and increasing excitatory postsynaptic currents in prefrontal cortex [35].

Glycogen synthase kinase-3 (GSK-3) may be also involved in the underlying mechanism of ketamine's antidepressant effect. GSK-3 is another important protein in brain function. Inhibition of GSK-3 may have a mood stabilizing effect [41]. Ketamine can inhibit GSK-3 by increasing its phosphorylation in mouse model of depression [42]. Besides, in this animal study, ketamine's antidepressant effect was absent in GSK-3 knock-in mice, whose GSK-3 activity was persistently active [42].

The antidepressant effect of ketamine may be also through modulating cortical GABA (*γ*-aminobutyric acid) levels. Decreased GABA levels are found in the anterior cingulate of patients with MDD [43]. In an animal study, administration of ketamine can blunt the depression-like behavior and increase GABA levels in the anterior cingulate following unpredictable stress [44]. In one clinical study, ketamine injection was associated with increased GABA/water ratio [45]. However, inconsistent results were found in different studies about the association between these neurotransmitter alternations and the antidepressant response of ketamine [45].

There are still some concerns about applying ketamine to clinical practice in the treatment of depression. First, strong evidence of treatment efficacy of ketamine is still lacking, especially for the long-term outcome [46]. Besides, the administration routes of ketamine in current studies are usually through intravenous injection which will limit its clinical use [46]. Most important of all, the safety issues of long-term ketamine administration, such as the risk of psychotomimetic effect, cognitive impairment, abuse, and dependence, need to be clarified in further investigations [47].

Since ketamine has a risk of psychotomimetic effects, such as dissociative state, other NMDA receptor antagonists are developed as potential antidepressant. AZD6765 (lanicemine) is a nonselective NMDA receptor antagonist which may have an antidepressant effect with a better safety profile. In a double-blind, randomized, crossover, placebo-controlled trial, a single infusion of AZD6765 produced rapid but short-lived antidepressant effect without producing psychotomimetic effects. The duration of antidepressant effect of AZD6765 in this study was only 110 minutes [48]. In another study, the antidepressant effect of single-dose AZD6765 peaked at 72 hours postinfusion and disappeared by 10–13 days after infusion [49]. This study also found that multiple infusions of AZD6765 (3 weeks treatment with an interval of three infusions per week) can sustain antidepressant effect to 5 weeks after the last infusion [49]. However, AZD6765 failed to show its efficacy in treatment-resistant MDD in phase II clinical trials [3].

### 2.2. Selective NMDA Receptor Subtype 2B (NR2B) Antagonists

Another way to develop NMDA receptor antagonists with more specificity and possibly better safety profile is targeting at specific subtypes of NMDA receptors. Among all NMDA receptor subtypes, NMDA receptor subtype 2B (NR2B) might be the most suitable candidate because NR2B subunit only expresses in the forebrain and it is associated with NMDA neurotoxicity [50]. In an animal study, mice with NR2B subunit knockdown in the bed nucleus of the stria terminalis had similar behavior as the affective effect after ketamine treatment [51]. Furthermore, genetic deletion of NR2B from principal cortical neurons in mice blocked ketamine's antidepressant effect, including suppression of depression-like behavior, increasing mTOR activation, and synaptic protein synthesis [52]. Therefore, several selective NR2B antagonists are being investigated as potential antidepressants.

CP-101, 606 (traxoprodil) is one of the selective NR2B antagonists. A single dose of CP-101, 606, like ketamine, can enhance synaptic activity in rat hippocampus. This indicates its potential antidepressant effect [53]. In a double blind, randomized, controlled clinical trial, a single infusion of CP-101, 606 had better antidepressant effect than placebo and this response can maintain for at least one week [54]. Unfortunately, further development of this compound was stopped because of QTc prolongation [3].

MK-0657 is the first oral form selective NR2B antagonist developed as an antidepressant [55]. In a pilot study, MK-0657 monotherapy (4–8 mg/d for 12 days) can significantly decrease the depressive symptoms compared to placebo in patients with treatment-resistant depression [55]. More studies are needed to validate the antidepressant effect of MK-0657.

Ro25-6891 is another NR2B antagonist showing potential antidepressant effect in some preclinical studies. Ro25-6891 can activate mTOR signalling [36]. Pretreatment with Ro25-6981 can prevent the acute stress-facilitated long-term depression in rat hippocampus [56]. Ro25-6981 also showed antidepressant effect in the behavior model of depression in rats [57]. Applying Ro25-6891 to clinical use is still under investigation.

### 2.3. NMDA Partial Agonists

NMDA partial agonists are usually seen as NMDA receptor modulators because they have agonist effect at low doses, but become antagonists at high doses. Therefore, NMDA partial agonists are investigated as potential antidepressants.

D-Cycloserine is one of the NMDA partial agonists. D-Cycloserine can restore impaired long-term potentiation in neural cell adhesion molecule-deficient mice model [58] and facilitate NMDA receptor-mediated synaptic potentials in rat hippocampal slices [59]. D-Cycloserine also increases expression of the activity-regulated cytoskeletal (Arc) protein, which was associated with memory consolidation [60]. It failed to produce antidepressant effect at dose 250 mg/d as adjuvant therapy for treatment-resistant MDD [61]. However, at high dose (1000 mg/day), D-cycloserine was effective as an add-on treatment for treatment-resistant depression [62].

GLYX-13 is another NMDA glycine-site functional partial agonist which has a potential antidepressant effect. A single infusion of GLYX-13 can sustain its antidepressant effect for 7 days in rat study [63]. In the same study, the authors also found that GLYX-13 could facilitate long-term potentiation, increase the proportion of whole-cell NMDA receptor current, and increase mature spine density in the brain [63]. The antidepressant effect of GLYX-13 may not only be related to NMDA receptor but also rely on AMPA/kainate receptor activation. In an animal study, pretreatment with AMPA receptor antagonist blocked GLYX-13's antidepressant effect [64]. Currently, the use of GLYX-13 in the treatment for depression is under phase II trial [64].

Sarcosine is a natural compound with activity of NMDA partial agonist. Sarcosine can improve depressive symptoms in both rodent models and patients with MDD. In a 6-week randomized, double-blinded, citalopram-controlled trial, sarcosine had better treatment response and less adverse effect than citalopram [65]. In an animal study, sarcosine decreased depressive-like behavior in forced swim test in rat and activated mTOR signalling pathway [66]. In the following study, sarcosine also increased mTOR signalling pathway activation and enhanced AMPA receptor membrane insertion in rodent model [67].

### 2.4. Glutamate Release Inhibitors

Other compounds involved in glutamatergic system are also being investigated as potential antidepressants. Riluzole, a glutamate release inhibitor, has multiple effects in glutamatergic system. In addition to inhibiting glutamate release, riluzole increases glutamate reuptake, blocks NMDA receptor activity, and increases AMPA receptor trafficking [3]. In animal model of depression, treatment of riluzole decreased hyperemotional response and improved depressive-like behavior [68, 69]. Besides, under higher dosage (60 *μ*g/ml), riluzole can restore hippocampal BDNF expression and increase glutamate glial transporter 1 expression [69]. In some pilot studies with small sample size, riluzole was effective in improving depressive symptoms in patients with depression [70, 71], but more rigorous study with larger sample size is needed to prove its antidepressant effect.

### 2.5. Metabotropic Glutamate Receptor Antagonist

In addition to NMDA receptor, metabotropic glutamate receptor (mGlu) may be another target for treating depression. LY341495 and MGS0039, which are competitive nonselective orthosteric mGlu2/3 receptor antagonists, have antidepressant-like effects in the animal model of depression [72, 73]. Coadministration of subeffective dose of LY341495 can enhance the antidepressant effect of scopolamine [74] and ketamine [75] in forced swimming test. In addition to rapid antidepressant effect, the antidepressant action of MGS0039 can sustain 3–7 days after a single-dose injection [72]. RO4491533, an mGluR2/3 negative allosteric modulator, reduces depression-like behavior in rodents [75]. Recently, a novel mGlu2/3 receptor antagonist which belongs to bicyclo[3.1.0]hexane glutamic acid analogs shows antidepressant-like effect in forced swimming test of mice [76]. The clinical studies of these mGlu 2/3 receptor antagonists including MGS0039 and RO4995819 are still under investigation [73].

mGlu5 receptor is another target for developing potential antidepressant. Basimglurant (RG7090, RO4917523) is a selective mGlu5 negative allosteric modulator with potential antidepressant activity and excellent drug-like properties [77]. In a phase 2b, double blind, randomized, placebo-controlled clinical trial, basimglurant (0.5 mg or 1.5 mg once daily) was adjunctive to antidepressant for six weeks. Although adjunctive basimglurant did not have significant difference to placebo in the major outcome (the change of clinician-rated of Montgomery-Asberg Depression Rating Scale (MADRS) score) [78], there were significant improvements in the 1.5 mg/d group in secondary outcomes, including patient-rated MADRS score, quick inventory of depressive symptomatology—self-report, clinical global impression—improvement mean score, and patient global impression—improvement mean score [78]. DSR-98776, another mGlu5 negative allosteric modulator, also shows antidepressant effect in rodent model of depression [79].

## 3. Brain Stimulation in the Treatment of Depression

In addition to medication, brain stimulation is another method to treat depressive disorders. Impaired neurocircuitry activity and reduced neuroplasticity are found in depressive disorders and play important roles in the pathophysiology of depression [6, 80–82]. Depressive patients had decreased motor-evoked potentials compared to normal subjects in paired associated stimulation [80]. Impaired connectivity in prefrontal cortex and anterior cingulate gyrus was related to the pathogenesis of depressive symptoms [6].

Brain stimulation therapy may exert its antidepressant effect by modulating neuroplasticity [83, 84]. Electroconvulsive therapy (ECT) which is used in treating depression refractory to medication is among the oldest brain stimulations. Several synaptic plasticity-associated transcripts and their encoding proteins in the hippocampus were affected by ECT [85]. In animal studies, microtubule-associated protein 2 in the dentate gyrus [86] and two endocytosis-related scaffolding proteins were increased after electroconvulsive seizure, and the authors implied that neurotransmitter transport trafficking may be involved in the therapeutic effect of ECT [87]. Hippocampal connectivity or volume of patients with MDD had been reported to be increased after receiving ECT in several clinical studies [88–92]. In addition to hippocampus, ECT can stimulate neurogenesis in frontal brain area in animal model [93] and modulate white matter microstructure in pathways connecting frontal and limbic areas in patients with MDD [94]. However, the association between these changes of neuroplasticity and depressive symptoms was still unclear [95, 96].

ECT also influences glutamatergic system. In depression model of rat, glutamate content was decreased and NR2B expression was upregulated following ECT [97]. Combining ketamine and ECT may have synergic effect in antidepressant effect. In a retrospective study, patients receiving ECT with ketamine needed fewer ECT sessions and had better antidepressant treatment response and cognitive function than those receiving ECT with thiopental (an anaesthetic agent) as a comparator [98].

### 3.1. Transcranial Magnetic Stimulation (TMS)

Since ECT has some side effects such as cognitive deficits, safer brain stimulation modalities are developed for the treatment of depressive disorders. Repetitive transcranial magnetic stimulation (rTMS) is a Food and Drug Administration- (FDA-) approved treatment for patients with MDD who are resistant to antidepressant treatment [99]. Transcranial magnetic stimulation (TMS) uses an electromagnetic coil on the scalp to create an alternating magnetic field. This magnetic field induces a secondary electric current in the brain without interference from the skin, muscle, and bone. According to the frequency of magnetic pulse administration, TMS can be divided into many types. rTMS is a kind of TMS given magnetic pulses repetitively for duration from seconds to minutes [100].

The mechanism about how TMS works on depression is still unclear. Like ECT, TMS may stimulate neurogenesis [101]. In a small clinical study, left amygdala volume was increased following TMS and this change was associated with the antidepressant treatment response [102]. TMS may also modulate brain activity and neurotransmitters, such as serotonin and dopamine [103]. One hypothesis is that depressive patients have impaired modulation of cortical excitability and TMS may alter this imbalance [104]. Evidences from functional imaging indicate that reduction in prefrontal cortex activation may be related to the treatment effect of TMS [105]. Recently, another study showed that TMS may exert its antidepressant effect by modulating functional connectivity between the central executive work and default mode network [106].

Current paradigm of TMS is based on previous studies, but the most effective protocol of TMS which should include precise position, optimal amplitude, frequency, and course is still under investigation [100]. In order to increase the treatment response, minimize individual variability, and avoid potential adverse effects of standard rTMS, some new protocols have been developed. Theta burst stimulation (TBS) is designed for reducing administration duration. It only requires 1 to 3 minutes of stimulation [107]. Some studies showed that TBS has similar or better efficacy in treating depression compared to rTMS [107]. Low-field synchronized transcranial magnetic stimulation (sTMS) tries to achieve clinical response under much lower energy than conventional rTMS. sTMS delivers stimulation at individual's alpha frequency (IAF) and uses brain's natural resonance at the IAF [108]. Although sTMS can improve depressive symptoms as an add-on treatment for MDD [109], no treatment efficacy is found in sTMS as a monotherapy for the treatment of MDD [110].

### 3.2. Transcranial Direct Current Stimulation (tDCS)

Transcranial direct current stimulation (tDCS) is another method of noninvasive brain stimulation for the treatment of depressive disorders. The device of tDCS has two electrodes, anode and cathode. tDCS applies a constant low current (0.5–2 mA) directly to the brain via these electrodes on the scalp and changes the cortical excitability. The brain area underlying anode becomes hyperexcitable, whereas the area underlying cathode becomes less excitable [111]. A noninferiority, triple-arm, placebo-controlled trial showed that tDCS was similarly effective to escitalopram in treating depression [112]. Meta-analysis also revealed that tDCS was significantly superior to sham group for all outcome measures in depression treatment [113]. Furthermore, not only improving depressive symptoms, tDCS also increases paired associative stimulation-induced neuroplasticity [114]. However, the results of following studies investigating treatment efficacy of tDCS were disappointing. In a randomized, sham controlled study, a 5-day session of tDCS did not improve depressive symptoms in treatment-resistant depression [115]. A systematic review article indicates that tDCS had better response and remission rate than the control group, but the difference was not significant statistically [111]. More studies are needed to elucidate the antidepressant effect of tDCS.

### 3.3. Cranial Electrotherapy Stimulation (CES)

Cranial electrotherapy (CES) applies pulsed, low amplitude electrical currents (usually less than 1 mA) to the brain via scalp electrodes. CES has been approved for the treatment of anxiety, depression, and insomnia from Food and Drug Administration in the United States [116]. Clinically, CES can decrease comorbid depression in anxiety disorders [117]. However, the Cochrane library review indicates that methodologically rigorous studies to examine the antidepressant effect of CES in the treatment of acute depression are lacking [118]. How CES exert its antidepressant effect is still unknown. CES may affect limbic system, reticular activating system, and the hypothalamus [119]. A recent study showed that CES could deactivate cortical brain activity and alter connectivity in default mode network [120]. How CES modulate underlying neuroplasticity or signalling pathway needs further investigation.

### 3.4. Magnetic Seizure Therapy (MST)

Magnetic seizure therapy (MST) is a new variant of TMS. The rationale of this therapy is based on ECT. It uses high-intensity rTMS to evoke seizures like ECT but with better control. The treatment effect of MST in depression is still under study [104]. In previous studies, the response rate of MST for depression was about 50–60% [121]. The mechanism of MST is still unclear. In a positron emission tomography study, the relative glucose metabolism was increased in the basal ganglia, orbitofrontal cortex, medial frontal cortex, and dorsolateral prefrontal cortex after receiving a treatment course of MST [122]. This implies that these regional brain activities may be related to the mechanism of treatment effect of MST. More studies are needed to investigate the MST effect on neuroplasticity or signalling.

## 4. Conclusion

MDD is a complex mental disorder. Effective clinical treatment strategy with favourable adverse effect profile is still lacking until now. Neurotransmission via NMDA receptors may be a new target for the treatment of depression. Ketamine has a rapid antidepressive effect, but its long-term efficacy and safety raises concerns and still needs further investigation. Other NMDA receptor and glutamate modulators also show antidepressive effects in small-scale studies. Future studies with more rigorous design and in larger scale are needed to validate their efficacy and safety. In addition to medication, noninvasive brain stimulation is another treatment strategy for MDD. Developing and standardizing the most effective and safest protocol are the key points in the future.

The discussion of the underlying neurobiological mechanisms of the aforementioned treatments for depression is based on the theory of neuroplasticity impairment. Current evidences seem to imply that dysfunction of neurotransmission might be only a tip of iceberg in the pathophysiology of depressive disorders. Signalling pathways, such as mTOR signalling and their effect on downstream synaptogenesis, synaptic plasticity, neurotransmission, and functional connectivity are keystones in the genesis of depressive disorders. This indicates a novel direction in the future development of antidepressive treatment.
